# Synergistic Effects of Devulcanized Rubber, Polyethylene, and Fumed Silica on the Rheological and Structural Stability of Bitumen

**DOI:** 10.3390/polym18020208

**Published:** 2026-01-12

**Authors:** Akkenzhe Bussurmanova, Anar Akkenzheyeva, Viktors Haritonovs, Remo Merijs-Meri, Janis Zicans, Uzilkhan Yensegenova, Yerzhan Imanbayev, Yerbolat Ayapbergenov, Maira Turkmenbayeva, Murshida Aimova, Assiya Boranbayeva, Martynas Jankauskas, Romans Kornisovs

**Affiliations:** 1 Pedagogy Faculty, Yessenov University, 32 Microdistrict, Aktau 130003, Kazakhstan; akkenzhe.bussurmanova@yu.edu.kz (A.B.); murshida.aimova@yu.edu.kz (M.A.); assiya.boranbayeva@yu.edu.kz (A.B.); 2 Engineering Faculty, Yessenov University, 32 Microdistrict, Aktau 130003, Kazakhstan; maira.turkmenbayeva@yu.edu.kz; 3 Faculty of Civil and Mechanical Engineering, Riga Technical University, 6A Kipsalas Street, LV-1048 Riga, Latvia; viktors.haritonovs@rtu.lv (V.H.); romans.kornisovs@rtu.lv (R.K.); 4 Faculty of Natural Sciences and Technology, Riga Technical University, 3 Paula Valdena Street, LV-1048 Riga, Latvia; remo.merijs-meri@rtu.lv (R.M.-M.); janis.zicans@rtu.lv (J.Z.); 5 Faculty of Chemistry and Chemical Technology, Al-Farabi Kazakh National University, Al-Farabi Avenue 71, Almaty 050040, Kazakhstan; uzilkhan.yensegenova@yu.edu.kz; 6 Laboratory of Petrochemical Processes, Republican State Enterprise “Institute of Combustion Problems”, Bogenbay Street, 172, Almaty 050012, Kazakhstan; erzhan.imanbayev@mail.ru; 7 Branch of Limited Liability Partnership “KazMunaiGas Engineering” “KazNIPImunaigas”, 35 Microdistrict, Section 6/1, Aktau 130000, Kazakhstan; e.ayapbergenov@kmge.kz; 8 Joint-Stock Company “Polylema”, Jegaines Street 8, Biruliskes, LT-54469 Kaunas, Lithuania; info@polylema.lt

**Keywords:** bitumen binder, rheological properties, physicomechanical properties, storage stability, elastic recovery

## Abstract

This study examines the influence of virgin polyethylene (vPE), recycled polyethylene (rPE), and Aerosil (A) on the performance of bitumen binders modified with partially devulcanized rubber (DVR). The experimental program included morphology analysis, determination of devulcanization degree, dynamic viscosity measurements, shear stress–shear rate analysis, load–displacement (*F–*Δ*l*) testing, storage-stability evaluation, ring and ball softening point (R&B), penetration (*P*), and elastic recovery (*ER*) testing. The results show that DVR-rPE-modified bitumen binders exhibit 20–35% higher viscosity and up to 25% greater elongation at the break compared to DVR-vPE-modified bitumen systems, indicating more effective interaction with the bitumen matrix. The incorporation of Aerosil increased viscosity ca. 1.5–2 times for DVR-rPE and DVR-vPE-modified systems, respectively. Meanwhile, top and bottom differences in R&B decreased by a factor of 1.6–5 for DVR-rPE and DVR-vPE-containing composites, respectively, demonstrating significant enhancement in structural stability during storage. Mechanical testing further revealed that DVR-rPE + A binders absorbed 10–20% more deformation energy and consistently maintained ER values above 70–80%, corresponding to a higher elastic recovery grade at 25 °C. Overall, the DVR-rPE + A system provided the most balanced improvements in rheological, mechanical, and thermal properties, confirming its potential for use in high-performance, thermally stable, and environmentally sustainable bituminous materials for pavement applications.

## 1. Introduction

Sustainability has become a global priority in the construction industry, promoting the use of waste materials to reduce environmental impact. Numerous innovative materials and technologies have been developed for road infrastructure to enhance durability and ensure resource efficiency, including the integration of waste plastics and rubber into the bituminous materials. According to the World Business Council for Sustainable Development and the Tire Industry Project, the large-scale generation of end-of-life tires necessitates their effective recycling [1,2,3]. The incorporation of rubber and plastic waste in the production of modified bitumen is recognized as an effective resource-saving and environmentally protective approach [4,5,6].

The continual growth in vehicle numbers increases the demand for durable and cost-effective road surfaces. Although modern polymer-modified binders enhance the strength and service life of asphalt concrete, their use can raise the overall mix cost by up to 40% [7,8]. Consequently, the partial replacement of virgin materials with rubber and plastic waste is emerging as a practical and environmentally sustainable solution. Rubber crumb has long been employed to modify bitumen, improving heat resistance, elasticity, and pavement fatigue life. Studies have demonstrated that rubber–bitumen mixtures can reduce reflective cracking and traffic noise while enhancing adhesion and skid resistance [9,10]. However, these binders exhibit high viscosity, require elevated processing temperatures, consume significant energy, and necessitate continuous mixing to prevent phase separation [11].

To address these challenges, researchers have explored the use of additives and the devulcanization of rubber. Devulcanization induces structural rearrangement of polymer chains, reducing viscosity and enhancing compatibility with bitumen [12]. However, the mechanical properties of bitumen modified with devulcanized rubber (DVR) remain debated, highlighting the need for further investigation [13,14]. In addition to rubber recycling, increasing attention has been directed toward the incorporation of plastic waste, primarily polyethylene and polypropylene, into bituminous road materials. These polymers improve rutting resistance and high-temperature stiffness but can adversely affect bitumen performance at low temperatures [15]. Low-density polyethylene (LDPE) exhibits better compatibility with bitumen due to its branched molecular structure [16]. Nevertheless, the low-temperature performance and storage stability of PE-modified asphalt have long posed significant challenges. Blending PE with ethylene–vinyl acetate containing a high vinyl acetate content has been shown to enhance compatibility between PE and bitumen [17].

In recent years, research has increasingly focused on hybrid modifiers combining devulcanized rubber and plastic waste. Studies indicate that such combined rubber–plastic systems exhibit a synergistic effect, enhancing both thermal and storage stability [18,19,20,21,22,23,24,25,26]. Chemical compounds generated during devulcanization—such as plasticizers, compatibilizers, and crosslinking agents—may improve phase stability in systems containing plastics [20,21,24]. The authors of [25] optimized a dry method for the combined incorporation of tire crumb and plastic, demonstrating a synergistic improvement of mechanical and rheological properties as well as the enhanced water resistance of the mixtures. Similarly, [22] reported reduced viscosity and a stabilized structure of the modified binder when crumb rubber and polyethylene were used together. Research has also shown that the addition of hybrid modifiers increases bitumen’s resistance to both high and low temperatures; however, increasing the proportion of rubber relative to plastic can reduce high-temperature performance, as a higher rubber content decreases the stability of the binder during elevated-temperature storage [24].

Despite advances in modifying petroleum bitumen with recycled rubber and plastic waste, several scientific and technological challenges remain unresolved. The incorporation of rubber crumb, DVR, and its hybrids enhance the binder properties; however, industrial applications are constrained by issues related to phase stability and long-term durability.

Ensuring storage stability over extended periods remains a critical challenge, as segregation arises from differences in density and chemical composition between the bitumen matrix and polymer additives. Since the recognition of phase separation in modified asphalt binders in the 1980s, various approaches have been proposed to evaluate storage stability, including tube tests, X-ray analysis, microscopy, and spectroscopy [27,28,29,30,31,32]. Among these, the tube test has become the most widely adopted, as it closely replicates the actual storage conditions of modified bitumen [27,33,34,35,36,37].

In recent years, it has been demonstrated that the incorporation of nanoparticles into rubber–polymer-modified bitumens significantly enhances storage stability and improves their physical and mechanical properties [38,39,40,41,42,43]. Fumed silica nanoparticles (FSNPs) function as a microstructural stabilizer: their branched and networked structure interacts with the binder matrix, preventing phase separation and forming a reinforced spatial network. When combined with the asphaltene–resin matrix and polymer chains (e.g., SBS or rubber components), FSNPs increase viscosity, improve elastic recovery, and enhance thermal stability. Studies indicate that FSNPs enhance high-temperature performance without substantially compromising low-temperature flexibility. For instance, Zhou et al. [27] reported that FSNPs form a branched network, resulting in a 2–3-fold increase in high-temperature stability and a 1.2–3-fold increase in fatigue strength. Similarly, Shi and Zhou [40] demonstrated that adding 2–6% FSNPs to a terminally mixed rubber–bitumen formulation (CR 30–50%) significantly improves elasticity, deformation resistance, and adhesion, while causing only minimal reductions in low-temperature performance. Research findings [42] indicate that incorporating 5% polymer in combination with nanomaterials (nanosilica and nanoclay) enhances the performance properties of bitumen, both before and after aging, leading to reduced residual deformation and improved low-temperature crack resistance. Furthermore, the authors of study [43] reported that the addition of nanomontmorillonite exerted a significant positive influence on the storage stability of rubber-crumb-modified binders.

The scientific novelty of this study lies in the integrated use of devulcanized rubber, virgin or recycled polyethylene, and the Aerosil to develop a novel bitumen binder with improved flow behavior and increased strength and storage stability. Unlike conventional SBS or rubber crumb systems, the proposed DVR–rPE + A formulation provides balanced viscoelastic behavior through a multi-faceted modifying effect, combining improved compatibility between bitumen and rPE with increased stability due to the structuring effect of Aerosil. The practical significance of this work is underscored by its high potential for industrial application, enabling the recycling of substantial volumes of waste tires and post-consumer plastic while simultaneously enhancing the strength, thermal stability, and fatigue life of asphalt concrete pavements.

## 2. Materials and Methods

### 2.1. Materials

B70/100-grade bitumen (“CASPI BITUM” JV LLP, Aktau, Kazakhstan) was employed for the preparation of modified bitumen. The physicochemical properties of the petroleum road bitumen (grade B70/100) are summarized in Table 1.

**Figure 1:**
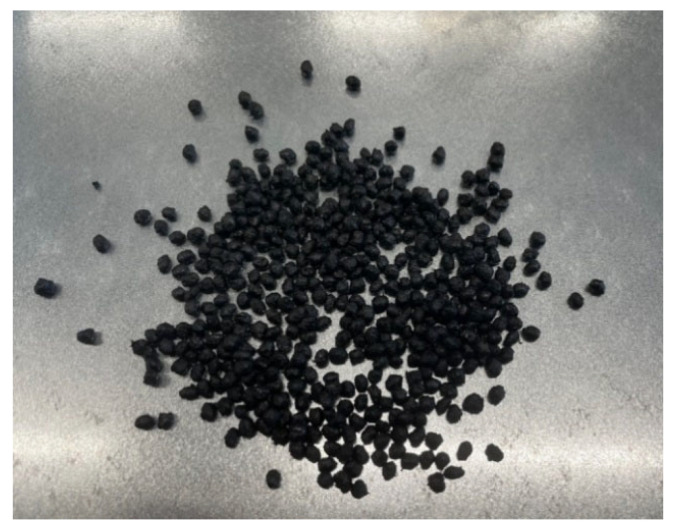
Devulcanized rubber compound as supplied by “Rubbintec” LLC, Jurmala, Latvia.

Customized partially devulcanized rubber (DVR) material supplied by “Polylema” JSC was utilized (Figure 1). “Rubbintec” LLC has developed a devulcanization method for rubber that employs a selective catalyst that is capable of decomposing sulfide bonds at relatively low temperatures while preserving the majority of the macromolecular chains [48]. The initial particle size of the rubber crumb for development of DVR was 0.08 mm.

During manufacturing, DVR was modified either with virgin polyethylene (vPE) or recycled polyethylene (rPE) to obtain a DVR compound, which was later used to modify the bitumen. The granules are black in color and consist of the following substances:

1. Crushed rubber from worn rubber tires 70–94%;
2. Polyethylene (vPE or rPE), 1–23%;
3. Additives 5–12%.

Aerosil 200 (A) from SIA Prestol Kompozits, Riga, Latvia, was chosen because in polymer–rubber–bitumen modification studies, the addition of Aerosil (fumed silica) has been shown to act as a rheology modifier and stabilizing agent: it forms a nanoscopic network that increases binder viscosity, improves dispersion of polymers and rubber particles, inhibits phase separation during storage, and enhances mechanical stability, elasticity, and temperature resistance in the modified bitumen [49].

### 2.2. Development of Modified Bitumen

The modified road bitumen binder was produced using a high-shear mixer, model L5M-A, Silverson Machines, Inc., East Longmeadow, MA, USA. Before mixing, the bitumen was pre-heated to 140 °C and poured in the mixing vessel located in the oil bath, after which a specified amount of the DVR compound modifier was gradually added. The mixing process proceeded for 60 min at 4500 rpm and 180 °C. In addition, a specified amount of the Aerosil was added to certain compositions during the mixing.

### 2.3. Characterization

Imaging procedures were conducted in accordance with EN 13632 [50], which specifies the method for the visualization of polymer distribution by fluorescent microscopy and emphasizes strict adherence to sample preparation and imaging conditions to ensure the comparability of fluorescence observations. Fluorescence imaging was performed using a Moticam ProS5 Lite fluorescence microscopy system (Motic Europe S.L.U., Barcelona, Spain). The cross-sectional area of the test specimens was examined under fluorescence excitation, using a D480/30 nm bandpass filter to assess the spatial distribution and morphological characteristics of fluorescent targets.

The sol fraction (*SF*) and gel fraction (*GF*) of the waste tire rubber crumb, as well as vPE- or rPE-modified DVR in duplicate were determined by Soxhlet extraction, using toluene as the solvent. Approximately 1.5 g of the analyzable material was packed in a dense mesh fabric and extracted for 24 h. After extraction, the samples were dried at 80 °C for 24 h to remove the residual solvent and weighed. *SF* was calculated as the ratio of the mass loss during extraction, defined as the difference between the initial mass (*W_1_*) and the mass after extraction (*W_2_*), to the initial mass (*W_1_*), as shown by Equation (1), whereas *GF* was calculated according to Equation (2).

(1) $$SF=\frac{W_{1}-W_{2}}{W_{1}}\times 100\%$$

(2) GF=100−SF

The crosslink density was determined by swelling the crosslinked rubber network in a solvent that does not disrupt the network. The swelling of waste tire rubber crumb as well as vPE- or rPE-modified DVR was conducted in toluene at room temperature for 72 h, followed by drying to a constant mass at 70 °C. The crosslink density (*Vₑ*, mol/cm^3^) was calculated using the Flory–Rehner Equation (3) [51,52].

(3) $$V_{e}=\frac{-[\ln\left(1-V_{r}\right)+V_{r}+\chi V_{r}^{2}]}{\left[V_{1}(V_{r}^{\frac{1}{3}}-V_{r}/2)\right]}$$

where *V_1_* is the molar volume of toluene (106.13 cm^3^ mol^−1^), *χ* is the rubber–solvent interaction parameter (*χ* = 0.39) [53], and *Vᵣ* is the volume fraction of rubber in the swollen sample. The value of *Vᵣ* was calculated using the Ellis–Welding Equation (4) [51,54].

(4) $$V_{r}=\frac{\frac{m_{r}}{\rho_{r}}}{\frac{m_{r}}{\rho_{r}}+\frac{m_{s}}{\rho_{s}}}$$

where *mₛ* is the mass of the swollen sample (g), *mᵣ* is the mass of the dry sample (g), $\rho_{s}$ is the density of toluene (0.8669 g/cm^3^), and $\rho_{r}$ is the density of the rubber sample.

Then, 70/100 and the modified bituminous binders were evaluated using the needle penetration test, softening point test, force ductility test, viscosity test, and the multiple stress creep and recovery (MSCR) test. Detailed descriptions of these testing procedures are provided in the following paragraphs.

The penetration test was conducted in accordance with EN 1426 [44], to assess the consistency of the bituminous binders and evaluate their susceptibility to deformation under applied loads at standard conditions. The experiments were conducted with a Matest B056-01 semi-automatic digital penetrometer (Matest S.p.A., Arcore, Italy). The standard deviation of the experiment was on average ±5%.

The softening point (*R&B*) test was conducted in accordance with EN 1427 [45], with two parallel measurements to evaluate the temperature susceptibility of the bituminous binders and assess their resistance to elevated temperatures. The experiments were conducted with the Matest B070M Softmatic automatic Ring and Ball apparatus (Matest S.p.A., Arcore, Italy), using glycerol as the test medium. The standard deviation of the experiment was, on average, ±2%.

The force ductility (cohesion) of the binders was conducted in accordance with EN 13589 [55], using appropriate dumbbell-shaped test specimens. The tests were made with a Matest B055-20M asphalt ductility tester (Matest S.p.A., Arcore, Italy) at 25 °C and a tensile speed of 50 mm/min. During the test, the tensile force and deformation were continuously recorded, and the deformation energy was calculated as the area under the force ductility–elongation curve.

The elastic recovery of the binders was studied using force ductility tests, which were carried out in accordance with EN 13398 [56]. The tests were made with a Matest B055-20M asphalt ductility tester (Matest S.p.A., Arcore, Italy) at 25 °C and a tensile speed of 50 mm/min. The elastic recovery was calculated according to the following Formula (5):

(5) $$ER=\frac{d}{L}\times 100$$

where *d*—distance between ends of the cut or broken test specimen, typically measured after 30 min. In the test, *L*—test specimen elongation (200 mm if the test specimen is not broken). From the resulting force–ductility curves, peak force and deformation were also determined.

The viscoelastic properties of the binders were characterized in accordance with ASTM D 4402-06 Standard Test Method for Viscosity Determination of Asphalt at Elevated Temperatures Using a Rotational Viscometer. Dynamic viscosity measurements at 180 °C were conducted using a Lamy DSR500 rotary viscometer (Lamy Rheology, Champagne au Mont d’Or, France) with the spindle/measuring system SV429.

Elastic recovery was evaluated using the multiple stress creep recovery (MSCR) test, in accordance with AASHTO TP 70 [57] and ASTM D7405 [58]. The MSCR test was conducted with an Anton Paar SmartPave 102 dynamic shear rheometer (DSR) (Anton Paar GmbH, Graz, Austria), with a 25 mm plate–plate configuration. The testing temperature was maintained at 64 °C, corresponding to the high-performance grade temperature of the neat binder. The procedure consisted of two loading phases at stress levels of 0.1 kPa and 3.2 kPa: 10 cycles at 0.1 kPa followed by 10 cycles at 3.2 kPa. The percentage recovery (*R*_0.1_ and *R*_3.2_) and non-recoverable creep compliance (*Jnr*_0.1_ and *Jnr*_3.2_) were calculated for both stress levels, where higher recovery percentages and lower *J_nr_* values indicate greater resistance to permanent deformation [59].

The widely used “tube test” was employed to assess storage stability following EN 13399 [60] by measuring the difference in softening point temperatures between the top and bottom sections of the aluminum tubes (32 mm diameter, 160 mm height). In this procedure, aluminum foil tubes of standard dimensions were carefully filled with hot binder to minimize air entrapment, sealed with a lid, and placed vertically in an oven at 163 ± 3 °C for 48 ± 1 h. After cooling at room temperature, the tubes were placed in a freezer for 2 h, then evenly sectioned into three parts and stored until testing at −20 °C. Before the subsequent tests, the binder samples from each of the sections were heated up to 140 °C, homogenized, and formed into the corresponding test specimens in accordance with appropriate testing standards, as described above.

Designations of the investigated modified bitumen compositions are summarized in Table 2.

## 3. Results and Discussion

### 3.1. Characterization of the Modifier

The structure of the developed bitumen modifier is revealed by determining the sol fraction (*SF*), gel fraction (*GF*), and cross-link density (*Vₑ*) of DVR modified with vPE or rPE, as is shown in Table 3.

**Figure 2:**
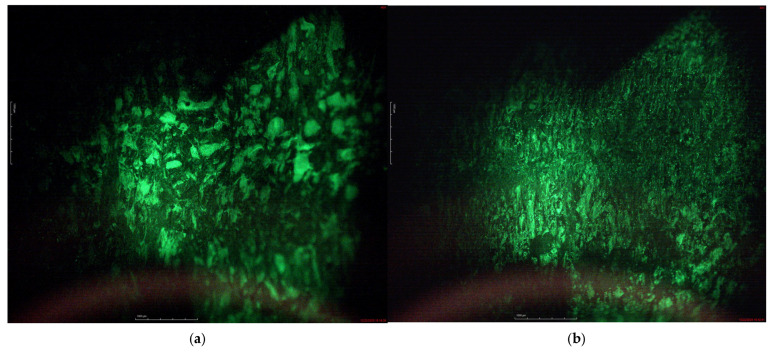
Fluorescent microscopy images of DVR-vPE (**a**) and DVR-rPE (**b**) modified bitumen compositions.

As is demonstrated in Table 3, *SF* of the tire rubber crumb is considerably lower and *GF* is higher in comparision with DVR modified with vPE or rPE, which confirms that the partial devulcanosation process has been caried out successfully. The *SF* and *GF* of the modified DVR specimens are rather similar; however, cross-linking degree of the rPE-modified DVR is larger, which may denote better devulcanisation behavior of tire rubber in the presence of rPE and customized devulcanisation additives. Interestingly, the calculated crosslink density for the rPE containing a DVR compound is larger than for its counterpart with vPE; this may be due to certain interaction between the partially oxidized recycled polymer and the DVR. This is demonstrated in images of fluorescent microscopy, taken from the cross-section of the modified bitumen sample (please see Figure 2). Fluorescence microscopy images reveal that bitumen forms the continuous matrix phase in which the unregularly shaped modifier particles are distributed, thus denoting heterogeneous/inhomogeneous systems complying with the first case, in accordance with EN 13632. In contradiction to the DVR-vPE, demonstrating comparatively large fluorescent modifier particles of varous sizes within the bitumen matrix, DVR-rPE-modified bitumen reveal a much finer morphology of tiny particles that are regularly distributed within the bitumen matrix. In addition, DVR-vPE particles represent broader particle size distribution in comparision to DVR-rPE, which denotes the larger agglomerration tendency of the former. The statement about good interaction between devulcanized rubber with bitumen is also confirmed by other researchers’ reports, e.g., the recent manuscript by Ma et al. [61].

### 3.2. Rotation Viscometry

**Figure 3:**
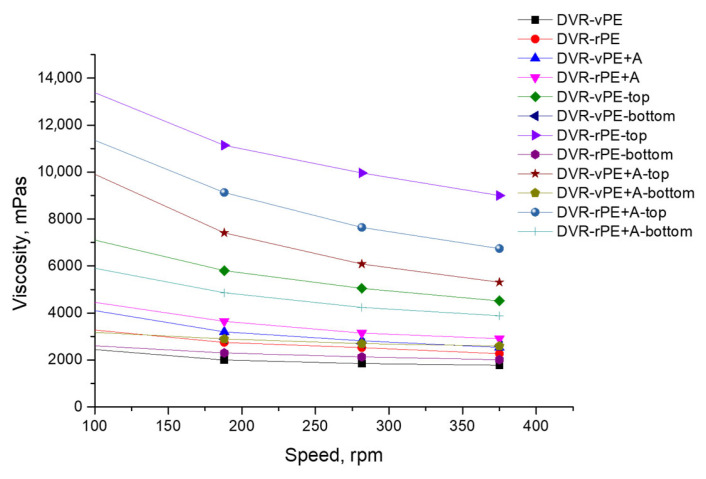
Shear viscosity–spindle rotation speed relationships of the investigated modified bitumen compositions.

In Figure 3, the shear viscosity–spindle rotation speed relationships are shown.

From here, the viscosity of the DVR-vPE-modified bitumen is the lowest of all the compositions investigated. This may be related to the lowest compatibility being between the modifier and the bitumen. In the presence of the Aerosil viscosity of the investigated bitumen systems before aging is increased in contradiction to the compositions without A, which may denote the structuring effect of A. After the storage stability test, the bitumen compositions with both DVR-rPE and DVR-rPE-A, taken from the top of the aged cylindrical test specimen, demonstrate the highest viscosity values, which may explain the concentration of the modifier in the upper part of the test specimen, due to density differences. Meanwhile, it is evident that after the A introduction in the DVR-rPE-modified bitumen, the viscosity difference between the virgin and the aged specimen is smaller than for the counterpart compositions without A. The same trend is also observed for DVR-vPE-A, though at smaller viscosity values.

**Figure 4:**
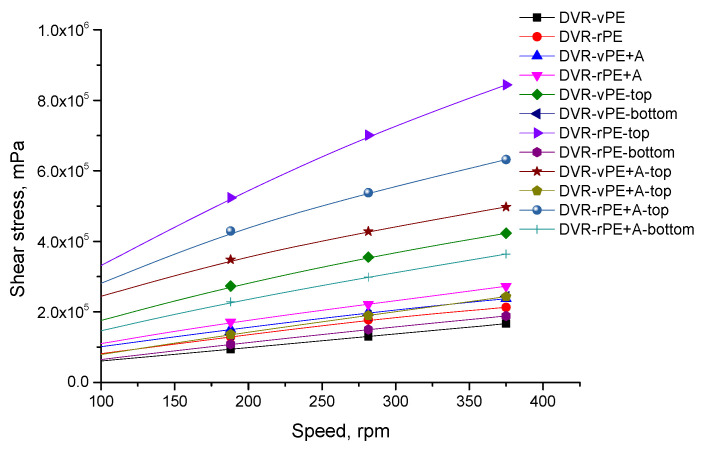
Shear stress–spindle rotation speed relationships of the investigated modified bitumen compositions.

In Figure 4, shear stress–shear speed relationships are shown.

The lowest shear stresses are determined to be for the DVR-vPE-modified bitumen composition. In the case of DVR-rPE bitumen, shear stresses are increased to a higher value at the final speed/shear rate value. After the storage stability test, a huge difference is observed between the “shear stress–speed/shear rate” relationships of the top and bottom parts of the aged DVR-rPE bitumen test specimens. However, after adding A, this difference is considerably reduced. In the case of DVR-vPE–bitumen compositions, the effect of the A addition is considerably smaller.

The obtained results indicate that the DVR-rPE compositions exhibit higher viscosity and shear strength compared with the DVR-vPE systems. This finding is consistent with the conclusions reported in reviews [62,63], which highlight the better compatibility of rPE with bitumen. The interaction between DVR, rPE, and A is governed by a combination of adsorption and interfacial processes occurring on the highly active surface of A. Polymer chains of DVR and rPE are adsorbed onto the A surface, mainly through Van der Waals interactions, as well as hydrogen bonding formed between functional groups of A and oxidized segments of rPE and DVR, generated during thermo-oxidative and photo-oxidative degradation of the parent materials. At the same time, particles of A tend to form a three-dimensional structural network, reducing the mobility of polymer-rich phases, preventing their migration, and significantly limiting vertical segregation within the bitumen matrix. This structural reinforcement enhances interfacial adhesion and improves the dispersion of DVR and rPE in bitumen, which manifests as increased rheological stability, reduced top–bottom variability after storage, and an improved elastic response and structural integrity of the modified binder. Similar mechanisms have been reported for nanosilica in polymer-modified asphalt systems [64,65].

A comparable increase in stiffness and an improvement in mechanical performance when using PE waste were also documented by the authors of the study [66]. The low viscosity and limited structural development observed in the vPE–bitumen systems in our work align with the reports on weak interfacial adhesion of virgin PE presented in [62,67].

The incorporation of dispersing additive A led to an increase in viscosity and a substantial reduction in modifier segregation during storage. This observation corresponds well to the results of other research groups [17,68,69], using different approaches to decrease segregation; for example, epoxidation of PE and addition of specific reactive systems, e.g., trans-polyoctenamer in conjunction with sulfur, applying peroxide crosslinking agents with silane coupling agents, etc. These studies demonstrate that such additives enhance interfacial adhesion, reduce phase separation, and improve the stability of PE-modified bituminous binders.

### 3.3. Softening Properties

**Figure 5:**
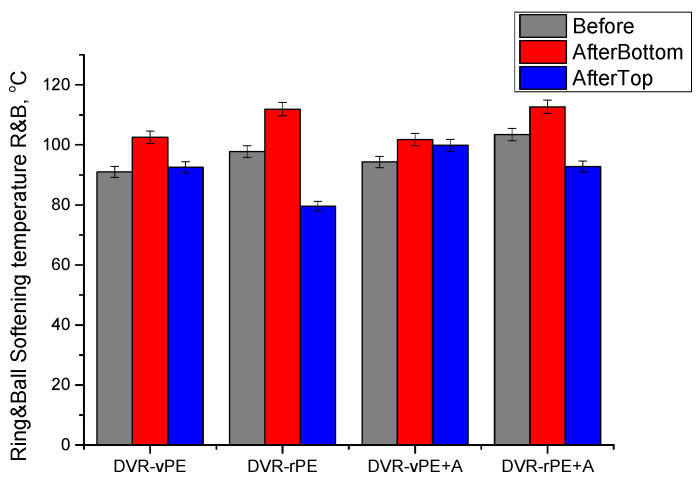
Ring and ball softening temperature of the investigated modified bitumen compositions.

In Figure 5, the ring and ball softening temperature (*R&B*) data of various DVR composites with bitumen are shown.

*R&B* values of DVR-vPE-modified bitumen compositions are 7 °C lower than those of DVR-rPE values, which may denote a higher stiffness of the modified bitumen, due to more efficient interaction of waste PE with bitumen. It is known that waste PE usually has a lower molecular weight, broader molecular weight distribution, and increased vinyl, vinylene, and oxygen-atom containing group content, due to the aging caused by the destruction of its macromolecular chain. The increased reactivity of rPE in the context of bitumen modification is an interesting issue from scientific and also practical viewpoints. This may increase the level of interaction between DVR-rPE with different bitumen fractions. Therefore, this aspect requires more in-depth investigation. Besides determining the destruction level of rPE and its correlation with macromolecular structure changes during the aging of different polyethylene products, this is of great practical interest to ensure a reliable supply chain of waste PE. At the moment, waste management companies are capable of delivering waste polyethylene at different aging stages from different sources, e.g., used agricultural film, used PE cans, etc. After the A addition, *R&B* is also increased by ca 3–5 °C for DVR-vPE- and DVR-rPE-modified bitumens, respectively. After the storage stability test, considerable differences in *ΔR&B* may be observed. The greatest differences in *ΔR&B* in the range (ca 32 °C) have been observed for rPE-containing systems, which is plausibly the effect of broader molecular weight distribution of the aged polymer. However, after the introduction of A during bitumen modification, the differences between the top and bottom parts of the aged modified bitumen compositions considerably decreased by ca 5 times for the DVR + vPE from 10 °C to 1.9 °C) and 1.5 times for the DVR + rPE (from 32.2 °C to 19.9 °C), demonstrating the stabilizing effect of A on the structure of the modified bitumen compositions.

**Figure 6:**
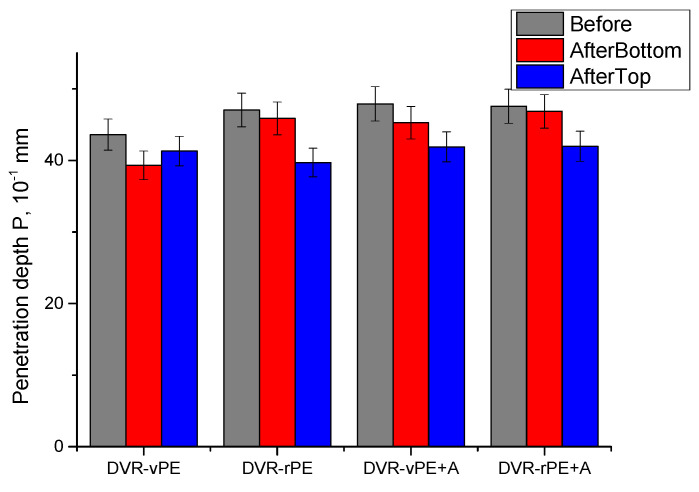
Penetration depth of the investigated modified bitumen compositions.

In Figure 6, the penetration depth, *P*, values for DVR-vPE, DVR-rPE, and DVR-modified bitumen are shown.

It may be observed that before aging, the *P* values of the DVR-rPE-modified bitumen compositions without A are somewhat higher than their counterparts with DVR-vPE (ca 5 units), which is interesting considering the R&B test values, while a stiffer binder should have higher R&B test values and lower penetration depth values. This, however, can be explained to some extent by considering the fact that the penetration test is more sensitive to small surface inhomogeneity of the composition. However, after the introduction of A into the bitumen mix, these differences considerably decrease, which may be explained by the improved homogeneity of the mixture because of the stabilizing effect of the dispersing aid. Interestingly, the effect of the A addition is more pronounced in the case of vPE-containing compositions, which may be explained by the fact that DVR-rPE-modified bitumen compositions initially have a better mix quality in comparison to DVR-vPE-containing systems. This may be due to better mixing of more reactive lower- and broader-molecular-weight rPE chains with DVR and bitumen compared to vPE chains. As expected, after the storage stability test, the difference in P between the bottom and top portions of the aged test cylinder is larger in the case of the DVR-rPE-modified bitumen when compared to the DVR-vPE-modified bitumen; meanwhile, the P of the bottom part demonstrates higher values, which is unusual. This, however, may demonstrate the fact that after aging, shorter molecular weight fragments of a relatively low-density DVR-rPE-modified bitumen composition concentrate in the upper part of the test specimen, thus making it stiffer in comparison to the bottom part. Interestingly, such behavior is also observed for A-modified compositions; however, this is to a lesser extent, disregarding whether rPE or vPE is used within DVR. It is also important that the difference between the bottom and top parts of the DVR-rPE-modified bitumen composition becomes smaller after A addition.

The softening temperatures of the DVR-rPE-modified bitumen systems obtained in this study are considerably higher than those of the DVR-vPE formulations. This trend is consistent with the findings reported in [17,63,70], which is attributed to the enhanced thermal performance, higher stiffness, and improved interfacial interactions of the recycled PE. An increase in the *R&B* softening point of DVR-rPE-modified binders has also been documented in [63], where PE waste contributed to improved deformation resistance and thermal stability. The slightly higher *P* values observed for the DVR-rPE-modified bitumen, despite their elevated *R&B* temperatures, align with the conclusions presented in [62], which emphasize the low sensitivity of the penetration test to local surface heterogeneities. The incorporation of the dispersing additive A reduced the differences in both *R&B* and *P* values between the upper and lower sections of the specimens after storage. This outcome is consistent with the stabilizing effects of the functional and silane-based additives reported in [17,64], which are known to enhance phase compatibility, suppress segregation, and improve storage stability in PE-modified bituminous binders.

In general, the change in *R&B* and *P* of the investigated compositions after thermal aging demonstrate that DVR with rPE are more suitable for bitumen modification.

### 3.4. Ductility

**Figure 7:**
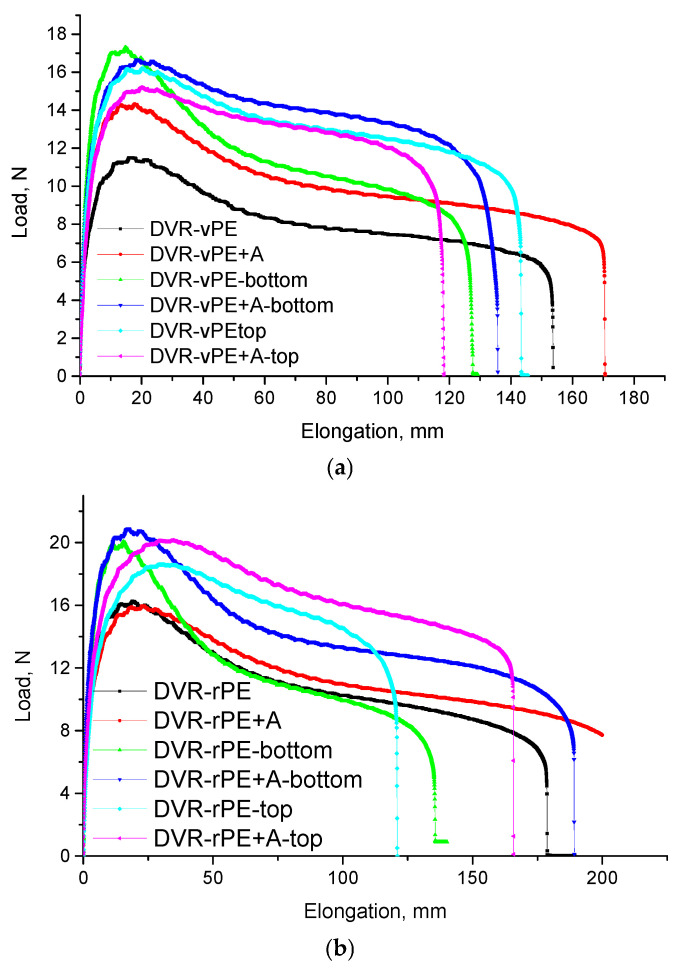
Load–displacement curves of the investigated DVR-modified bitumen compositions with vPE (**a**) and rPE (**b**).

Figure 7 depicts the load–displacement (*F–*Δ*l*) curves for DVR-vPE or DVR-rPE-modified bitumen compositions for clear comparison of their elastic recovery behavior. It is demonstrated that all the investigated modified bitumen compositions demonstrate the same type of *F–*Δ*l* curves with an expressed yielding maximum, following stable deformation until rupture or 200 mm. It is clearly demonstrated that before exposure to the storage stability test, the modified bitumen composition with DVR-rPE demonstrates a higher yield maxima and larger elongation at break values in comparison to those that are characteristic for the DVR-vPE-modified bitumen.

Such behavior may indicate that DVR-rPE interact with bitumen more efficiently in comparison to DVR-vPE, which may be explained with a typically lower molecular weight and broader molecular weight distribution of the recycled polymer, resulting in shorter macromolecular chains, which can more readily interact with bitumen. After the modification of A, a considerable improvement of the modified bitumen compositions is observed, disregarding the chosen polymer (vPE or rPE). In the case of DVR-vPE-modified bitumen compositions after the addition of A yield maxima and elongation at the break, both are increased by ca 30% and 10%, respectively. Although in the case of DVR-rPE-modified bitumen compositions, an increment of these parameters is less pronounced (increment of elongation at break at least by 14% and hardly changed the yield maxima), in general, after A addition, the absolute values of the rPE-containing system are still higher than those, which is characteristic for DVR-vPE-modified bitumen compositions. This is also clearly demonstrated in Table 4, where the characteristic absorbed deformation energy values of the investigated compositions are summarized.

As expected, after the storage stability test, *F–*Δ*l* behavior of the investigated compositions considerably change; in general, yield strength is increased, whereas deformation at the break is decreased, resulting in a subsequent change in the absorbed deformation energy values. In general, the higher absorbed energy values of DVR-rPE systems (in comparison to those of DVR-vPE) are evidently related to increased interaction between the aged polymer moieties with DVR and different bitumen fractions. After aging, DVR-rPE + A-modified bitumen demonstrated an increment of the absorbed energy, especially for the test specimens taken from the top part of the test tube sample, which may denote the concentration of the DVR-rPE-modified bitumen-rich phase in the upper part of the composition, as it was already marked before. However, it is important to mention that after the storage stability test, the absorbed energy value of the DVR-rPE + A-based bitumen specimen, taken from the bottom part of the test tube sample, was only slightly lower (ca 2%) than that taken from the top part of the test sample. Another interesting fact is the increased values for the absorbed energy after the storage stability test of DVR + rPE + A modified bitumen in comparison to the unmodified counterpart, which may denote that during high temperature storage (163 °C), additional interaction between DVR-rPE + A and different bituminous fractions occur. This fact, however, should be treated cautiously, while the absorbed energy value of the DVR-rPE + A test specimen before aging cannot be directly compared with other modified bitumen compositions because of its different recovery behavior, i.e., the test specimen was cut in contradiction to others, which ruptured at diverse deformation values, as demonstrated in Figure 6. Different deformation behavior, i.e., different values of elongation at the break, is probably the reason for the ambiguous change in the absorbed energy values after the storage stability test. For SBS-modified bitumen (containing 4–6% polymer by bitumen mass), the value of *N* was 3.5, which is 4–6 times lower than that observed for polyethylene-modified bitumen compositions. The elastic recovery at this relatively low force ranged from 60% to 80%.

**Figure 8:**
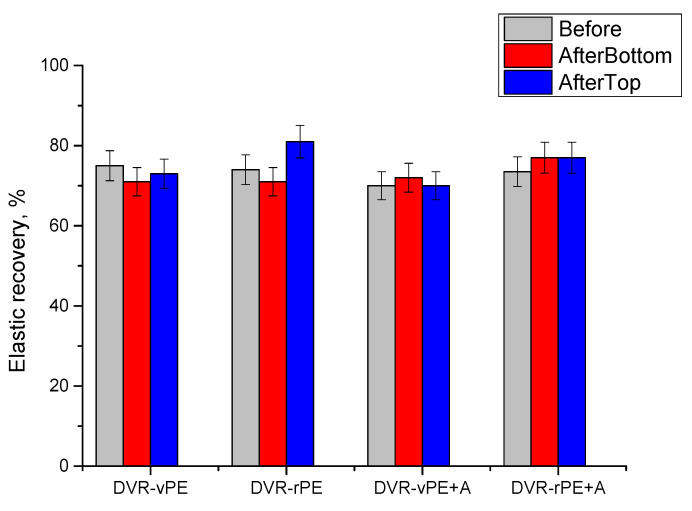
Elastic recovery of the investigated modified bitumen compositions.

In Figure 8, the elastic recovery (*ER*) values of the investigated modified bitumen compositions are shown. In general, for all the tested DVR-vPE- and DVR-rPE-modified bitumen compositions, the *ER* values are above 70, which indicates that the bitumen class is increased from the fifth (*ER* at 25 °C > 50%) to the third (*ER* at 25 °C > 70%) or even the second (*ER* at 25 °C > 80%). Concomitantly, the modified bitumen compositions based on DVR-rPE overall show similar or even higher elastic recovery values in comparison to those that are characteristic for DVR-vPE-modified bitumen systems. It is also evident that after A addition to DVR-vPE-modified bitumen, *ER* values slightly decrease, which may be explained with the addition of high surface area and high-modulus mineral filler, which usually decreases the elastic recovery of materials. Concomitantly, in the case of DVR-rPE + A, practically no difference in *ER* value is observed in comparison to the counterpart systems without A. Meanwhile, it is important that there is practically no difference in the *ER* between the top and bottom portions of the latter system (DVR-rPE + A), which demonstrates its greater stability in comparison to the other tested bituminous binder compositions.

The increased yield stress and elongation at the break observed for the DVR-rPE-modified bitumen systems are consistent with the findings reported in [63,70], which indicate that recycled PE exhibits higher reactivity and forms a more elastic, structured network due to its shorter and more polar chains. The higher absorbed energy values measured for the DVR-rPE-modified bitumen systems also correlate with the results of [64], where PE waste enhanced the strength and deformation resistance of the binder. The incorporation of the dispersing additive A into both the DVR-vPE- and DVR-rPE-based formulations further improved their strength and deformation characteristics. This trend agrees with the effects of the functionalized and silane additives described in [17,68], which were shown to promote better polymer dispersion and increase elastic recovery. The higher *ER* values (>70–80%) obtained for the DVR-rPE-modified bitumen systems in the present study also support the conclusions of [63], demonstrating that recycled PE provides greater elasticity and resistance to permanent deformation compared with virgin PE. The minimal differences between the upper and lower sections of the DVR-rPE + A specimens after storage indicate reduced phase segregation: an effect similar to that reported for stabilized PE/EVA systems in [17].

### 3.5. MSCR Test

**Figure 9:**
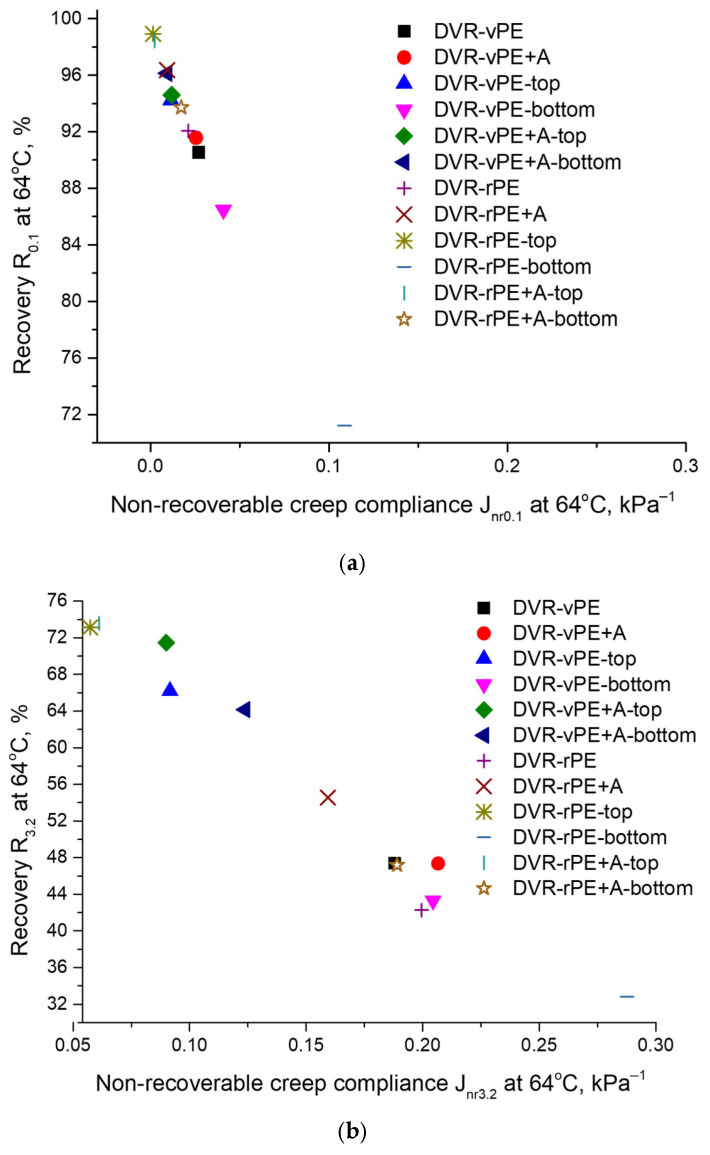
Results of the MSCR test at 64 °C for 0.1 kPa (**a**) and 3.2 kPa (**b**) load levels of the investigated modified bitumen compositions.

In Figure 9, the MSCR test results of the developed bitumen compositions are summarized.

It is shown that at 0.1 kPa loading, DVR-vPE- and DVR-rPE-modified bitumen compositions demonstrate similar elastic recovery, *R*, and non-coverable compliance, *J*, values that are close to 92% and 0.02 kPa^−1^, respectively. With the addition of the customized dispersion aid, *R*, the DVR-rPE-modified bitumen composition is considerably increased to 96%, whereas in the case of the DVR-vPE-modified bitumen, only slight changes are observed. After the storage stability test without A, both the DVR-vPE- and DVR-rPE-modified bitumen compositions demonstrate considerable differences between the top and bottom parts, with respect to the *R* and *J* values. However, after A addition, the difference between the top and bottom parts of the modified bitumen compositions is considerably reduced.

A similar trend is also observed at a higher loading level, i.e., at 3.2 kPa. Before aging, the systems without A demonstrate rather low *R* values and high *J* values, reaching ca 48% and ca 0.18 for DVR-vPE-modified bitumen systems and ca 42% and ca 0.20 for DVR-rPE-modified bitumen compositions. After A addition, for DVR-rPE-modified bitumen compositions, *R* is increased up to ca 55%, whereas *J* is decreased to 0.16 kPa^−1^. In its turn, after A addition to DVR-vPE-modified bitumen compositions, *R* remains practically unchanged, while *J* increases to 0.21 kPa^−1^. Thermal aging induced differences between the top and bottom parts of the storage stability test specimens, which were also larger for the investigated modified bitumen compositions without A.

The *R* values of approximately 92–96% and the *J* values of about 0.02 kPa^−1^ obtained at low stress levels for the DVR-PE-modified binders are consistent with the results reported in [68], where rPE-modified binders demonstrated enhanced elasticity, attributed to shorter polymer chains and improved compatibility. The increase in *R* after the incorporation of the additive A into the DVR-rPE-modified bitumen systems also aligns with the findings of [65,67], which showed that silane and reactive additives reduce residual deformation and lower *J* by improving interfacial adhesion. The pronounced differences between the upper and lower sections of the stored specimens in the formulations without the additive A correspond to the observations in [62], where PE–bitumen composites were reported to be prone to vertical segregation and phase separation.

At a stress level of 3.2 kPa, the decrease in *R* and the increase in *J* observed in our study are in agreement with the results of [63,64], which indicated that PE-modified binders exhibit limited elasticity under higher loading, especially in the absence of stabilizing additives. The subsequent increase in *R* and decrease in *J* after the addition of additive A to the DVR-rPE-modified bitumen systems fully correspond to the mechanisms described in [65,67], where active additives enhanced deformation recovery under elevated stresses. The minimized differences between the upper and lower layers of the DVR-rPE + A specimens after storage further confirm that additive A significantly improves structural stability and mitigates phase separation, which is consistent with the trends reported in [67].

In general, one may conclude that A positively influences the stability of the investigated modified bitumen compositions. Although at small loads (0.1 kPa), the *R* values of the DVR-based bitumen modifiers are very high (even 98% for DVR-rPE-modified bitumen in the presence of A), at higher loads (3.2 kPa), a significant reduction in elastic recovery occurs. Disregarding this, the addition of A during manufacturing of the investigated bitumen compositions positively influenced the stability of the DVR-based compositions independently, whether vPE or rPE was used for bitumen co-modification.

### 3.6. Techno-Economic and Practical Implications

The techno-economic assessment conducted for binder systems containing DVR, dispersing agent A, and either vPE (priced at 1100–1200 €·t^−1^) or rPE (priced at 600 €·t^−1^) indicates that the use of recycled polyethylene provides a clear cost advantage. As shown, the unit price of rPE is approximately 40% lower than that of vPE, while the cost contribution of the dispersing agent A remains limited, due to its low dosage.

Importantly, this cost reduction is accompanied by enhanced material performance after the addition of A, which exhibited superior rheological stability, improved homogeneity after storage, and a higher elastic recovery. These results highlight the relevance of polyethylene, and especially rPE, not only as a cost-efficient component (in comparison to a typically used SBS bitumen modifier (3000 EUR/t at least)) but also as a functional material that strengthens the structural integrity of modified binders. Moreover, the inclusion of recycled polymers (rPE, waste tire crumb) aligns with the sustainability objectives by reducing resource consumption and supporting circular-material pathways. Thus, especially the rPE–DVR–A system demonstrates strong potential for both practical implementation and environmentally responsible material design. Overall, the techno-economic and sustainability assessment confirms that the DVR-rPE + A system provides a highly favorable balance of performance, cost efficiency, and environmental impact. The use of recycled polymers reduces the material cost while improving the rheological stability and elastic behavior, and the LCA evidence demonstrates substantially lower embodied energy and CO_2_ emissions compared with vPE [71,72,73,74,75]. These combined advantages highlight the practical feasibility and ecological relevance of adopting rPE-based modification strategies for high-performance bituminous binders.

## 4. Conclusions

This study demonstrates that the performance of DVR-modified bitumen is strongly governed by the type of polyethylene used and by the incorporation of Aerosil. Recycled polyethylene showed consistently better compatibility with the bituminous matrix, resulting in increased stiffness, enhanced elasticity, and improved structural stability after thermal exposure. The addition of A further promoted homogeneity, reduced phase segregation during storage, and strengthened the rheological response of the modified binders. Due to the improved compatibility, the morphology of DVR-rPE-modified bitumen revealed finely sized particles that were more regularly distributed within the bitumen matrix.

The combined findings indicate that the DVR-rPE + A system provides the most balanced improvement across all evaluated properties. Rheological tests confirmed the stabilizing role of A, as reflected in the reduced differences between the upper and lower sections of the samples and in the overall strengthening of the internal structure. The softening point and penetration results revealed that DVR-rPE contributes better resistance to aging, while A enhances structural uniformity and mitigates property gradients caused by phase migration.

Mechanical tests further showed that DVR-rPE-modified bitumen compositions possess higher load-bearing capacity and elongation at the break compared with DVR-vPE-based systems, with the dispersing aid A improving these characteristics for both polymer types. Elastic recovery values remained above 70% for all DVR-vPE- and DVR-rPE-modified binders, and the negligible variation between the upper and lower parts of DVR-rPE + A samples highlights their excellent storage stability.

Overall, the results confirm that the combined use of recycled polyethylene, devulcanized rubber, and the dispersing aid A enables the development of high-performance bitumen binders with superior storage stability, absorbed energy, and elasticity. The DVR-rPE + A composition demonstrates the highest potential for practical applications requiring enhanced deformation tolerance and long-term structural integrity.

## Figures and Tables

**Table 1:** Physicochemical properties of petroleum road viscous bitumen (grade B70/100).

Bitumen Properties	Actual Value	Test Method
Penetration at 25 °C, 0.1 mm, not lower	87.2	EN 1426:2015 [44]
Softening point °C, not below	45.85	EN 1427:2015 [45]
Change in properties after RTFOT, in accordance with EN 12591:2009 [46]		
Change in Mass, %	−0.0212 (≤0.008) *	EN 12607-1:2015 [47]
Change in Softening Point °C	+7.0 (≤9) *	EN 1427:2015 [45]
Retained Penetration, %	51.9 (>46) *	EN 1426:2015 [44]

* In brackets, the minimum values of the retained properties of B70/100 in accordance with EN 12591:200 are denoted.

**Table 2:** Designations of the developed and investigated modified bitumen compositions.

Abbreviation	Compositions Based on vPE	Compositions Based on rPE
*Before Aging*		
vPE	virgin polyethylene	-
rPE	-	recycled polyethylene
DVR-vPE	70/100 modified with DVR-vPE	
DVR-rPE	-	70/100 modified with DVR-rPE
DVR-vPE + A	70/100 modified with DVR-vPE and A	-
DVR-rPE + A	-	70/100 modified with DVR-rPE and A
*After aging by storage stability test*		
DVR-vPE-top	Top part of 70/100 modified with DVR-vPE	-
DVR-vPE-bottom	Bottom part of 70/100 modified DVR-vPE	-
DVR-rPE-top	-	Top part of 70/100 modified with DVR-rPE
DVR-rPE-bottom	-	Bottom part of 70/100 modified with DVR-rPE
DVR-vPE + A-top	Top part of 70/100 modified DVR with vPE and A	-
DVR-vPE + A-bottom	Bottom part of 70/100 modified with DVR-vPE and A	-
DVR-rPE + A-top	-	Top part of 70/100 modified with DVR-rPE and A
DVR-rPE + A-bottom	-	Bottom part of 70/100 modified with DVR-rPE and A

**Table 3:** Solvent-affected structural parameters of the modifiers used in comparison to tire rubber crumb.

Material	Sol Fraction (SF), %	Gel Fraction (GF), %	Crosslink Density (mol/cm^3^ × 10^−5^)
Tire rubber crumb	10	90	5.72
DVR modified with vPE	52	48	0.69
DVR modified with rPE	56	44	1.46

**Table 4:** Absorbed energy of DVR-vPE/rPE-modified bitumen compositions during force ductility test.

	Before Storage Stability Test, J/cm^2^	After Storage Stability Test, Bottom, J/cm^2^	After Storage Stability Test, Top, J/cm^2^
**DVR-vPE**	1.266	1.497	1.879
**DVR-rPE**	1.978	1.681	1.897
**DVR-vPE + A**	1.738	1.864	1.521
**DVR-rPE + A**	2.288	2.692	2.736

## Data Availability

The original contributions presented in this study are included in the article. Further inquiries can be directed to the corresponding author.
